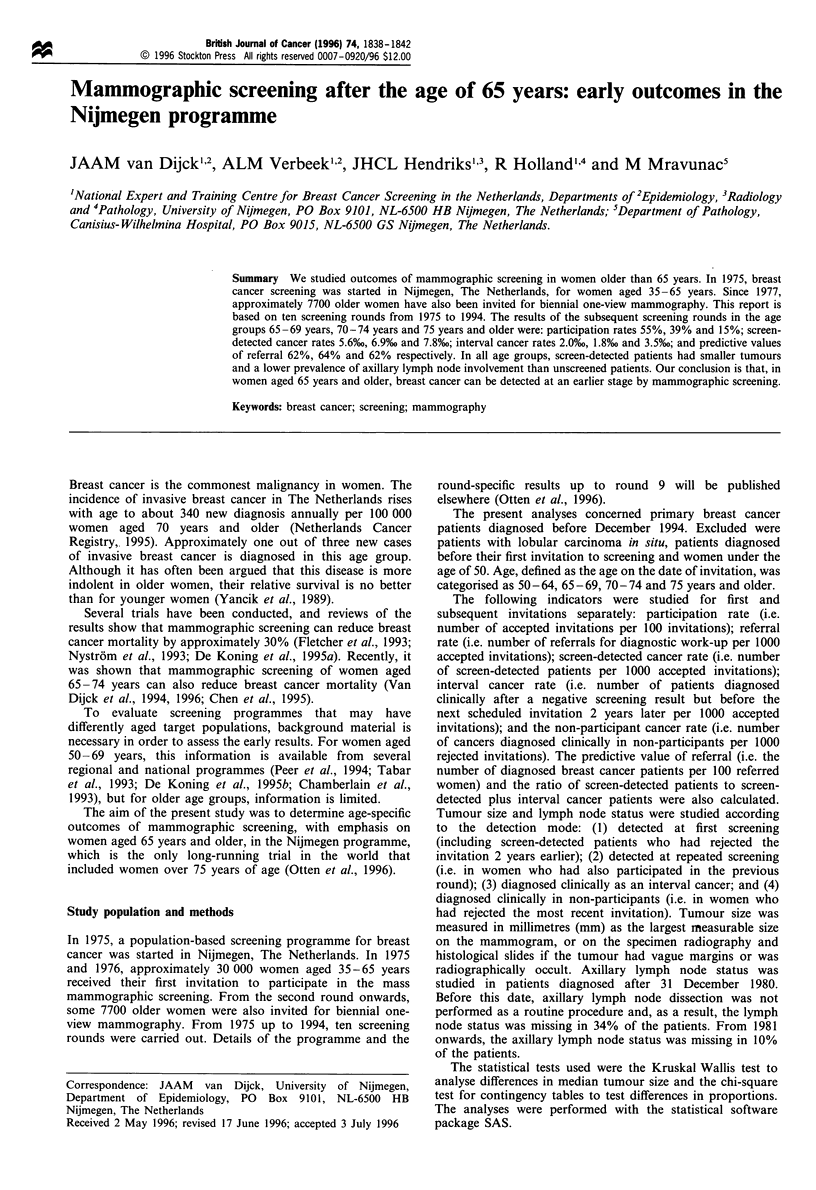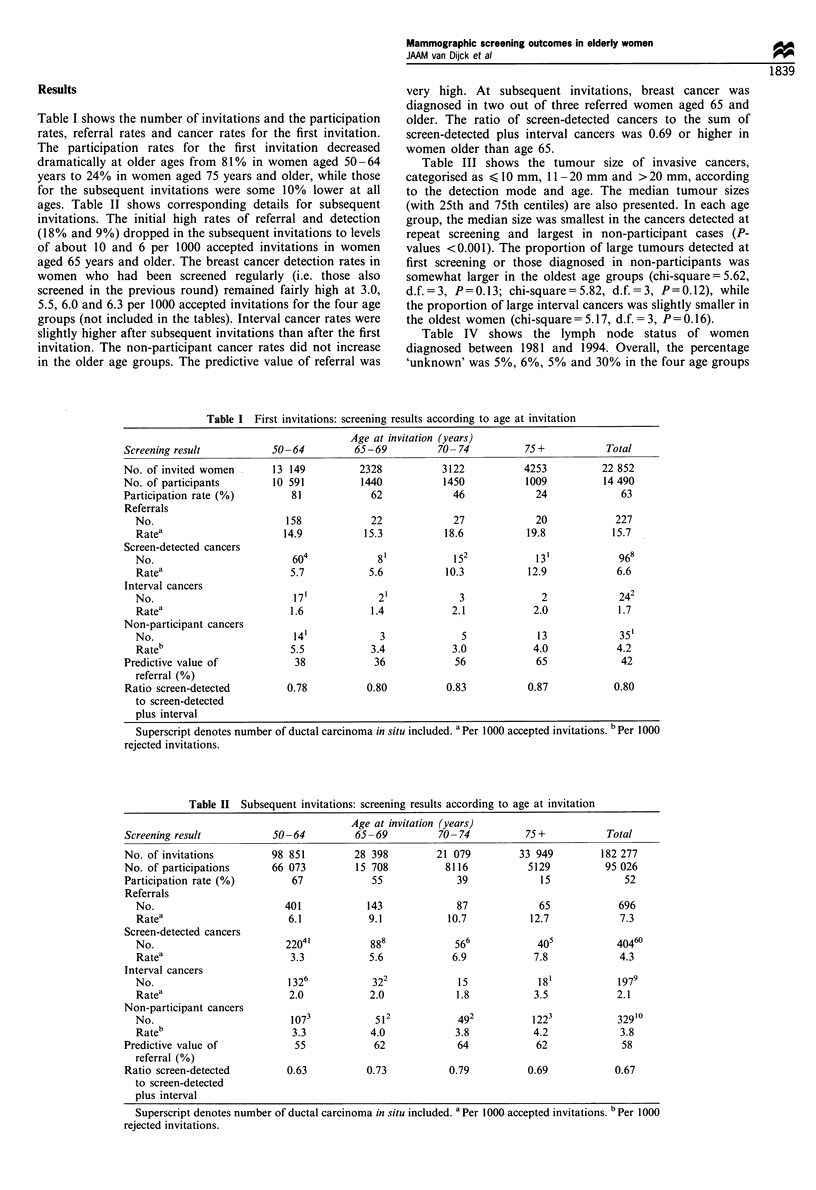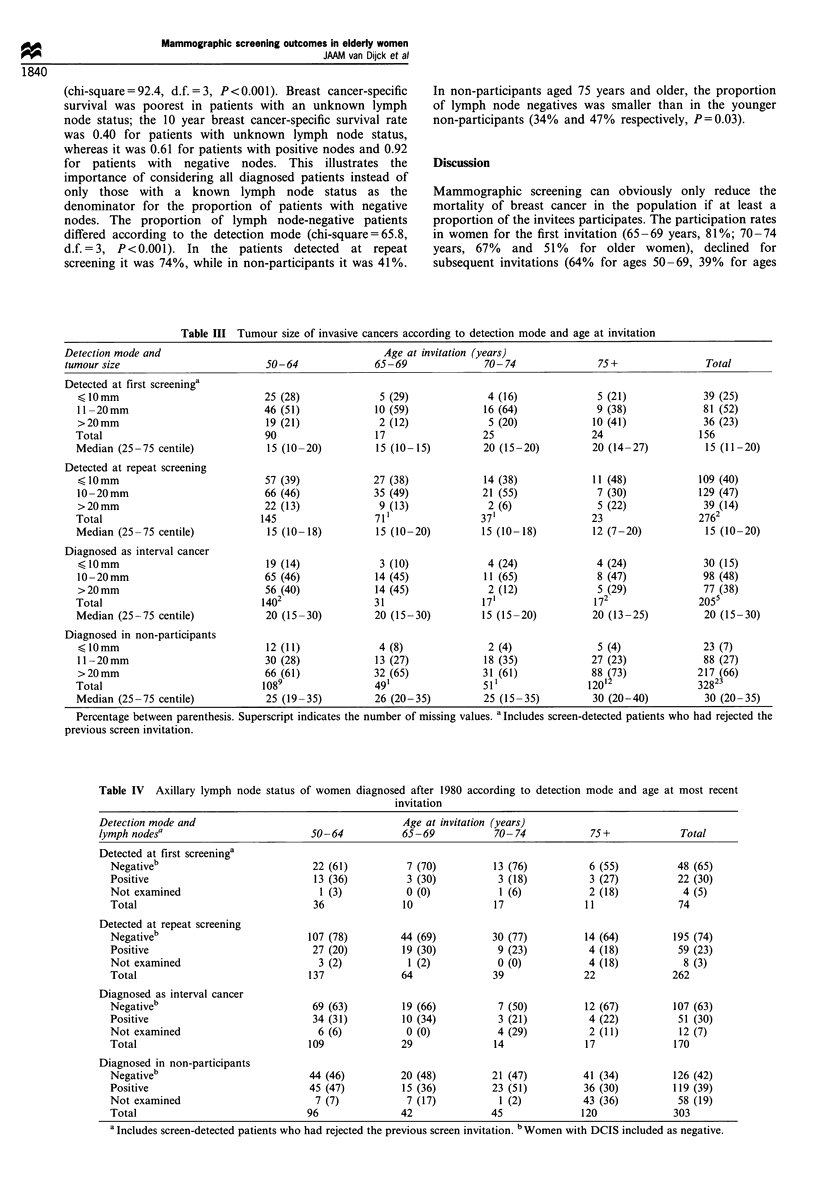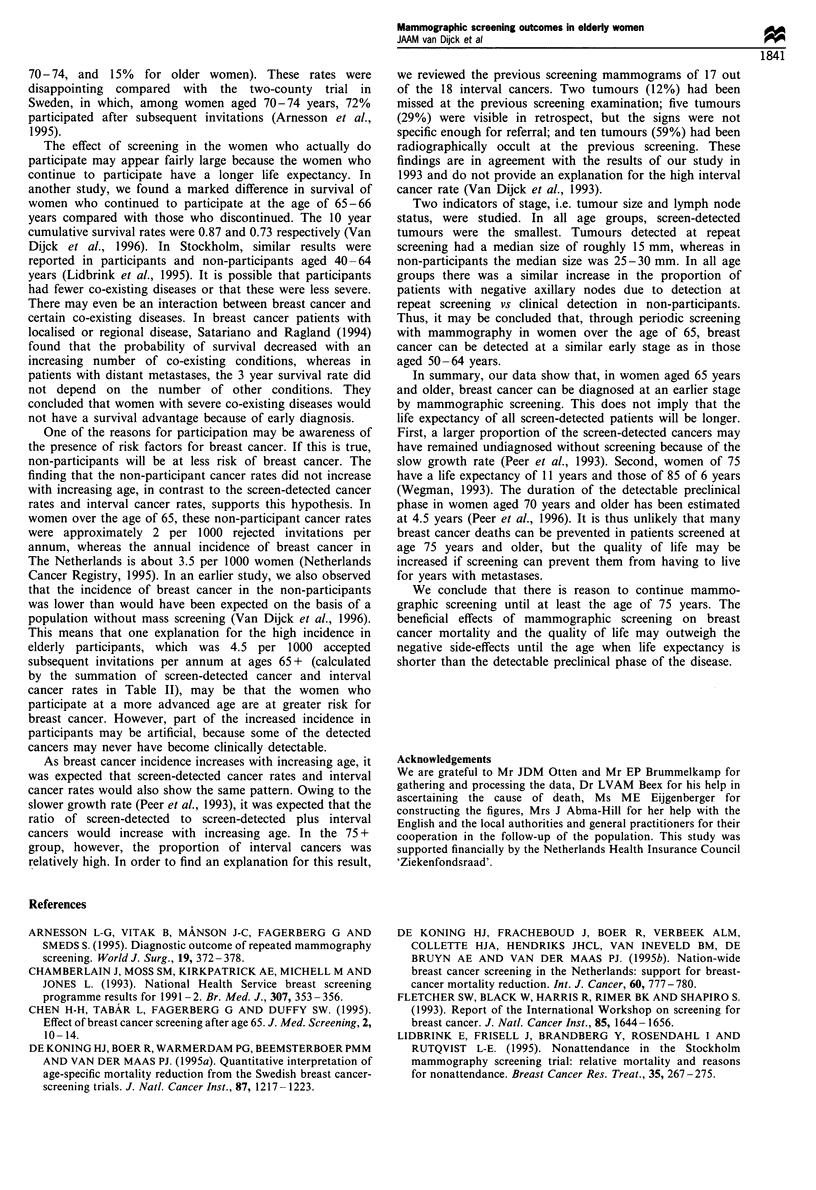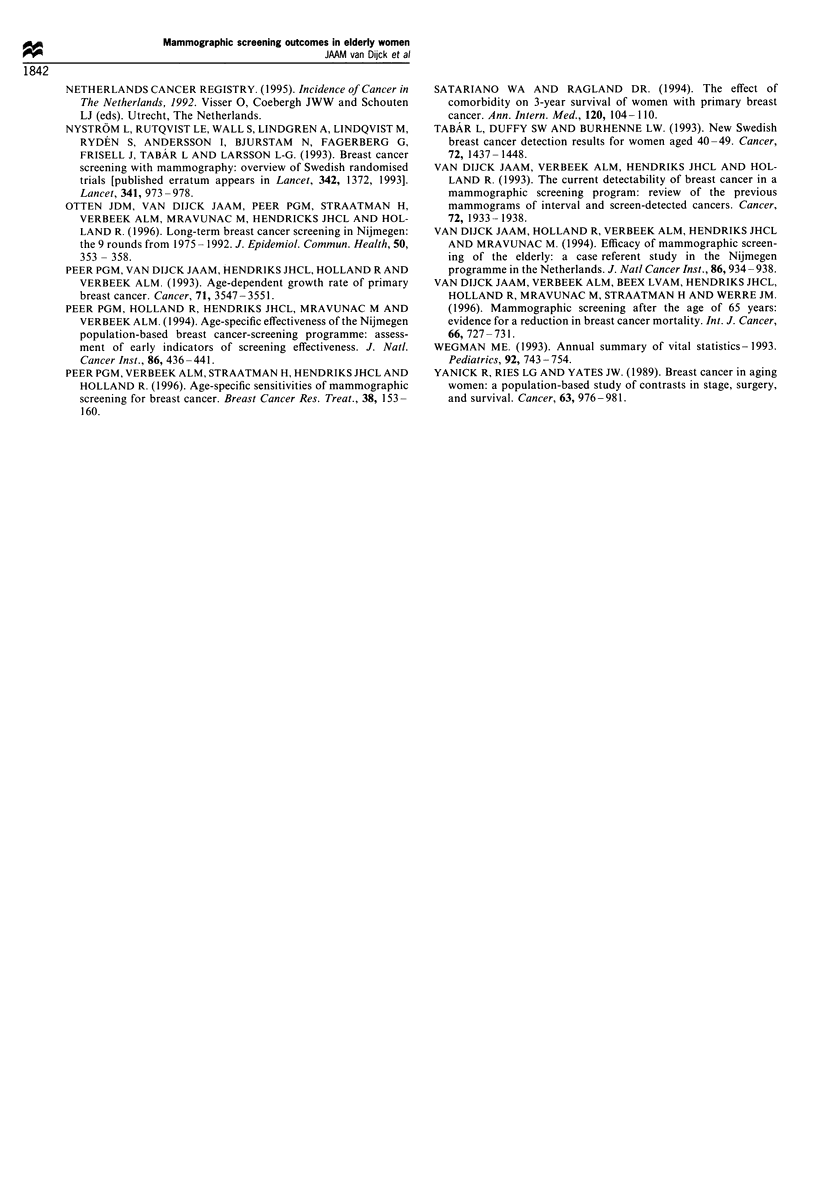# Mammographic screening after the age of 65 years: early outcomes in the Nijmegen programme.

**DOI:** 10.1038/bjc.1996.640

**Published:** 1996-12

**Authors:** J. van Dijck, A. Verbeek, J. Hendriks, R. Holland, M. Mravunac

**Affiliations:** Department of Epidemiology, University of Nijmegen, The Netherlands.

## Abstract

We studied outcomes of mammographic screening in women older than 65 years. In 1975, breast cancer screening was started in Nijmegen, The Netherlands, for women aged 35-65 years. Since 1977, approximately 7700 older women have also been invited for biennial one-view mammography. This report is based on ten screening rounds from 1975 to 1994. The results of the subsequent screening rounds in the age groups 65-69 years, 70-74 years and 75 years and older were: participation rates 55%, 39% and 15%; screen-detected cancer rates 5.6%, 6.9% and 7.8%; interval cancer rates 2.0%, 1.8%, and 3.5%; and predictive values of referral 62%, 64% and 62% respectively. In all age groups, screen-detected patients had smaller tumours and a lower prevalence of axillary lymph node involvement than unscreened patients. Our conclusion is that, in women aged 65 years, and older, breast cancer can be detected at an earlier stage by mammographic screening.


British Journal of Cancer (1996) 74, 1838-1842
© 1996 Stockton Press All rights reserved 0007-0920/96 $12.00

### Mammographic screening after the age of 65 years: early outcomes in the Nijmegen programme

JAAM van Dijck[1,2], ALM Verbeek[1,2], JHCL Hendriks[1,3], R Holland[1,4] and M Mravunac[5]

[1]*National Expert and Training Centre for Breast Cancer Screening in the Netherlands, Departments of* [2]*Epidemiology,* [3]*Radiology and* [4]*Pathology, University of Nijmegen, PO Box 9101, NL-6500 HB Nijmegen, The Netherlands;* [5]*Department of Pathology, Canisius-Wilhelmina Hospital, PO Box 9015, NL-6500 GS Nijmegen, The Netherlands.*

**Summary** We studied outcomes of mammographic screening in women older than 65 years. In 1975, breast cancer screening was started in Nijmegen, The Netherlands, for women aged 35–65 years. Since 1977, approximately 7700 older women have also been invited for biennial one-view mammography. This report is based on ten screening rounds from 1975 to 1994. The results of the subsequent screening rounds in the age groups 65–69 years, 70–74 years and 75 years and older were: participation rates 55%, 39% and 15%; screen-detected cancer rates 5.6‰, 6.9‰ and 7.8‰; interval cancer rates 2.0‰, 1.8‰ and 3.5‰; and predictive values of referral 62%, 64% and 62% respectively. In all age groups, screen-detected patients had smaller tumours and a lower prevalence of axillary lymph node involvement than unscreened patients. Our conclusion is that, in women aged 65 years and older, breast cancer can be detected at an earlier stage by mammographic screening.

**Keywords:** breast cancer; screening; mammography

Breast cancer is the commonest malignancy in women. The incidence of invasive breast cancer in The Netherlands rises with age to about 340 new diagnosis annually per 100 000 women aged 70 years and older (Netherlands Cancer Registry, 1995). Approximately one out of three new cases of invasive breast cancer is diagnosed in this age group. Although it has often been argued that this disease is more indolent in older women, their relative survival is no better than for younger women (Yancik *et al.*, 1989).

Several trials have been conducted, and reviews of the results show that mammographic screening can reduce breast cancer mortality by approximately 30% (Fletcher *et al.*, 1993; Nyström *et al.*, 1993; De Koning *et al.*, 1995*a*). Recently, it was shown that mammographic screening of women aged 65–74 years can also reduce breast cancer mortality (Van Dijck *et al.*, 1994, 1996; Chen *et al.*, 1995).

To evaluate screening programmes that may have differently aged target populations, background material is necessary in order to assess the early results. For women aged 50–69 years, this information is available from several regional and national programmes (Peer *et al.*, 1994; Tabar *et al.*, 1993; De Koning *et al.*, 1995*b*; Chamberlain *et al.*, 1993), but for older age groups, information is limited.

The aim of the present study was to determine age-specific outcomes of mammographic screening, with emphasis on women aged 65 years and older, in the Nijmegen programme, which is the only long-running trial in the world that included women over 75 years of age (Otten *et al.*, 1996).

### Study population and methods

In 1975, a population-based screening programme for breast cancer was started in Nijmegen, The Netherlands. In 1975 and 1976, approximately 30 000 women aged 35–65 years received their first invitation to participate in the mass mammographic screening. From the second round onwards, some 7700 older women were also invited for biennial one-view mammography. From 1975 up to 1994, ten screening rounds were carried out. Details of the programme and the round-specific results up to round 9 will be published elsewhere (Otten *et al.*, 1996).

The present analyses concerned primary breast cancer patients diagnosed before December 1994. Excluded were patients with lobular carcinoma *in situ*, patients diagnosed before their first invitation to screening and women under the age of 50. Age, defined as the age on the date of invitation, was categorised as 50–64, 65–69, 70–74 and 75 years and older.

The following indicators were studied for first and subsequent invitations separately: participation rate (i.e. number of accepted invitations per 100 invitations); referral rate (i.e. number of referrals for diagnostic work-up per 1000 accepted invitations); screen-detected cancer rate (i.e. number of screen-detected patients per 1000 accepted invitations); interval cancer rate (i.e. number of patients diagnosed clinically after a negative screening result but before the next scheduled invitation 2 years later per 1000 accepted invitations); and the non-participant cancer rate (i.e. number of cancers diagnosed clinically in non-participants per 1000 rejected invitations). The predictive value of referral (i.e. the number of diagnosed breast cancer patients per 100 referred women) and the ratio of screen-detected patients to screen-detected plus interval cancer patients were also calculated. Tumour size and lymph node status were studied according to the detection mode: (1) detected at first screening (including screen-detected patients who had rejected the invitation 2 years earlier); (2) detected at repeated screening (i.e. in women who had also participated in the previous round); (3) diagnosed clinically as an interval cancer; and (4) diagnosed clinically in non-participants (i.e. in women who had rejected the most recent invitation). Tumour size was measured in millimetres (mm) as the largest measurable size on the mammogram, or on the specimen radiography and histological slides if the tumour had vague margins or was radiographically occult. Axillary lymph node status was studied in patients diagnosed after 31 December 1980. Before this date, axillary lymph node dissection was not performed as a routine procedure and, as a result, the lymph node status was missing in 34% of the patients. From 1981 onwards, the axillary lymph node status was missing in 10% of the patients.

The statistical tests used were the Kruskal Wallis test to analyse differences in median tumour size and the chi-square test for contingency tables to test differences in proportions. The analyses were performed with the statistical software package SAS.

Correspondence: JAAM van Dijck, University of Nijmegen, Department of Epidemiology, PO Box 9101, NL-6500 HB Nijmegen, The Netherlands
Received 2 May 1996; revised 17 June 1996; accepted 3 July 1996

### Results

Table I shows the number of invitations and the participation rates, referral rates and cancer rates for the first invitation. The participation rates for the first invitation decreased dramatically at older ages from 81% in women aged 50–64 years to 24% in women aged 75 years and older, while those for the subsequent invitations were some 10% lower at all ages. Table II shows corresponding details for subsequent invitations. The initial high rates of referral and detection (18% and 9%) dropped in the subsequent invitations to levels of about 10 and 6 per 1000 accepted invitations in women aged 65 years and older. The breast cancer detection rates in women who had been screened regularly (i.e. those also screened in the previous round) remained fairly high at 3.0, 5.5, 6.0 and 6.3 per 1000 accepted invitations for the four age groups (not included in the tables). Interval cancer rates were slightly higher after subsequent invitations than after the first invitation. The non-participant cancer rates did not increase in the older age groups. The predictive value of referral was very high. At subsequent invitations, breast cancer was diagnosed in two out of three referred women aged 65 and older. The ratio of screen-detected cancers to the sum of screen-detected plus interval cancers was 0.69 or higher in women older than age 65.

Table III shows the tumour size of invasive cancers, categorised as $\leqslant 10$ mm, 11–20 mm and $>20$ mm, according to the detection mode and age. The median tumour sizes (with 25th and 75th centiles) are also presented. In each age group, the median size was smallest in the cancers detected at repeat screening and largest in non-participant cases ($P$-values $<0.001$). The proportion of large tumours detected at first screening or those diagnosed in non-participants was somewhat larger in the oldest age groups (chi-square = 5.62, d.f. = 3, $P=0.13$; chi-square = 5.82, d.f. = 3, $P=0.12$), while the proportion of large interval cancers was slightly smaller in the oldest women (chi-square = 5.17, d.f. = 3, $P=0.16$).

Table IV shows the lymph node status of women diagnosed between 1981 and 1994. Overall, the percentage ‘unknown’ was 5%, 6%, 5% and 30% in the four age groups

**Table I** First invitations: screening results according to age at invitation

| *Screening result* | *Age at invitation (years)* 50–64 | 65–69 | 70–74 | 75+ | *Total* |
|---|---|---|---|---|---|
| No. of invited women | 13 149 | 2328 | 3122 | 4253 | 22 852 |
| No. of participants | 10 591 | 1440 | 1450 | 1009 | 14 490 |
| Participation rate (%) | 81 | 62 | 46 | 24 | 63 |
| Referrals | | | | | |
| No. | 158 | 22 | 27 | 20 | 227 |
| Rate[a] | 14.9 | 15.3 | 18.6 | 19.8 | 15.7 |
| Screen-detected cancers | | | | | |
| No. | 60[4] | 8[1] | 15[2] | 13[1] | 96[8] |
| Rate[a] | 5.7 | 5.6 | 10.3 | 12.9 | 6.6 |
| Interval cancers | | | | | |
| No. | 17[1] | 2[1] | 3 | 2 | 24[2] |
| Rate[a] | 1.6 | 1.4 | 2.1 | 2.0 | 1.7 |
| Non-participant cancers | | | | | |
| No. | 14[1] | 3 | 5 | 13 | 35[1] |
| Rate[b] | 5.5 | 3.4 | 3.0 | 4.0 | 4.2 |
| Predictive value of referral (%) | 38 | 36 | 56 | 65 | 42 |
| Ratio screen-detected to screen-detected plus interval | 0.78 | 0.80 | 0.83 | 0.87 | 0.80 |

Superscript denotes number of ductal carcinoma *in situ* included. [a] Per 1000 accepted invitations. [b] Per 1000 rejected invitations.

**Table II** Subsequent invitations: screening results according to age at invitation

| *Screening result* | *Age at invitation (years)* 50–64 | 65–69 | 70–74 | 75+ | *Total* |
|---|---|---|---|---|---|
| No. of invitations | 98 851 | 28 398 | 21 079 | 33 949 | 182 277 |
| No. of participations | 66 073 | 15 708 | 8116 | 5129 | 95 026 |
| Participation rate (%) | 67 | 55 | 39 | 15 | 52 |
| Referrals | | | | | |
| No. | 401 | 143 | 87 | 65 | 696 |
| Rate[a] | 6.1 | 9.1 | 10.7 | 12.7 | 7.3 |
| Screen-detected cancers | | | | | |
| No. | 220[41] | 88[8] | 56[6] | 40[5] | 404[60] |
| Rate[a] | 3.3 | 5.6 | 6.9 | 7.8 | 4.3 |
| Interval cancers | | | | | |
| No. | 132[6] | 32[2] | 15 | 18[1] | 197[9] |
| Rate[a] | 2.0 | 2.0 | 1.8 | 3.5 | 2.1 |
| Non-participant cancers | | | | | |
| No. | 107[3] | 51[2] | 49[2] | 122[3] | 329[10] |
| Rate[b] | 3.3 | 4.0 | 3.8 | 4.2 | 3.8 |
| Predictive value of referral (%) | 55 | 62 | 64 | 62 | 58 |
| Ratio screen-detected to screen-detected plus interval | 0.63 | 0.73 | 0.79 | 0.69 | 0.67 |

Superscript denotes number of ductal carcinoma *in situ* included. [a] Per 1000 accepted invitations. [b] Per 1000 rejected invitations.

(chi-square = 92.4, d.f. = 3, $P<0.001$). Breast cancer-specific survival was poorest in patients with an unknown lymph node status; the 10 year breast cancer-specific survival rate was 0.40 for patients with unknown lymph node status, whereas it was 0.61 for patients with positive nodes and 0.92 for patients with negative nodes. This illustrates the importance of considering all diagnosed patients instead of only those with a known lymph node status as the denominator for the proportion of patients with negative nodes. The proportion of lymph node-negative patients differed according to the detection mode (chi-square = 65.8, d.f. = 3, $P<0.001$). In the patients detected at repeat screening it was 74%, while in non-participants it was 41%. In non-participants aged 75 years and older, the proportion of lymph node negatives was smaller than in the younger non-participants (34% and 47% respectively, $P=0.03$).

### Discussion

Mammographic screening can obviously only reduce the mortality of breast cancer in the population if at least a proportion of the invitees participates. The participation rates in women for the first invitation (65–69 years, 81%; 70–74 years, 67% and 51% for older women), declined for subsequent invitations (64% for ages 50–69, 39% for ages

**Table III** Tumour size of invasive cancers according to detection mode and age at invitation

| *Detection mode and tumour size* | *Age at invitation (years)* 50–64 | 65–69 | 70–74 | 75+ | *Total* |
|---|---|---|---|---|---|
| Detected at first screening[a] | | | | | |
| ⩽10 mm | 25 (28) | 5 (29) | 4 (16) | 5 (21) | 39 (25) |
| 11–20 mm | 46 (51) | 10 (59) | 16 (64) | 9 (38) | 81 (52) |
| >20 mm | 19 (21) | 2 (12) | 5 (20) | 10 (41) | 36 (23) |
| Total | 90 | 17 | 25 | 24 | 156 |
| Median (25–75 centile) | 15 (10–20) | 15 (10–15) | 20 (15–20) | 20 (14–27) | 15 (11–20) |
| Detected at repeat screening | | | | | |
| ⩽10 mm | 57 (39) | 27 (38) | 14 (38) | 11 (48) | 109 (40) |
| 10–20 mm | 66 (46) | 35 (49) | 21 (55) | 7 (30) | 129 (47) |
| >20 mm | 22 (13) | 9 (13) | 2 (6) | 5 (22) | 39 (14) |
| Total | 145 | $71^{1}$ | $37^{1}$ | 23 | $276^{2}$ |
| Median (25–75 centile) | 15 (10–18) | 15 (10–20) | 15 (10–18) | 12 (7–20) | 15 (10–20) |
| Diagnosed as interval cancer | | | | | |
| ⩽10 mm | 19 (14) | 3 (10) | 4 (24) | 4 (24) | 30 (15) |
| 10–20 mm | 65 (46) | 14 (45) | 11 (65) | 8 (47) | 98 (48) |
| >20 mm | 56 (40) | 14 (45) | 2 (12) | 5 (29) | 77 (38) |
| Total | $140^{2}$ | 31 | $17^{1}$ | $17^{2}$ | $205^{5}$ |
| Median (25–75 centile) | 20 (15–30) | 20 (15–30) | 15 (15–20) | 20 (13–25) | 20 (15–30) |
| Diagnosed in non-participants | | | | | |
| ⩽10 mm | 12 (11) | 4 (8) | 2 (4) | 5 (4) | 23 (7) |
| 11–20 mm | 30 (28) | 13 (27) | 18 (35) | 27 (23) | 88 (27) |
| >20 mm | 66 (61) | 32 (65) | 31 (61) | 88 (73) | 217 (66) |
| Total | $108^{9}$ | $49^{1}$ | $51^{1}$ | $120^{12}$ | $328^{23}$ |
| Median (25–75 centile) | 25 (19–35) | 26 (20–35) | 25 (15–35) | 30 (20–40) | 30 (20–35) |

Percentage between parenthesis. Superscript indicates the number of missing values. [a] Includes screen-detected patients who had rejected the previous screen invitation.

**Table IV** Axillary lymph node status of women diagnosed after 1980 according to detection mode and age at most recent invitation

| *Detection mode and lymph nodes[a]* | *Age at invitation (years)* 50–64 | 65–69 | 70–74 | 75+ | *Total* |
|---|---|---|---|---|---|
| Detected at first screening[a] | | | | | |
| Negative[b] | 22 (61) | 7 (70) | 13 (76) | 6 (55) | 48 (65) |
| Positive | 13 (36) | 3 (30) | 3 (18) | 3 (27) | 22 (30) |
| Not examined | 1 (3) | 0 (0) | 1 (6) | 2 (18) | 4 (5) |
| Total | 36 | 10 | 17 | 11 | 74 |
| Detected at repeat screening | | | | | |
| Negative[b] | 107 (78) | 44 (69) | 30 (77) | 14 (64) | 195 (74) |
| Positive | 27 (20) | 19 (30) | 9 (23) | 4 (18) | 59 (23) |
| Not examined | 3 (2) | 1 (2) | 0 (0) | 4 (18) | 8 (3) |
| Total | 137 | 64 | 39 | 22 | 262 |
| Diagnosed as interval cancer | | | | | |
| Negative[b] | 69 (63) | 19 (66) | 7 (50) | 12 (67) | 107 (63) |
| Positive | 34 (31) | 10 (34) | 3 (21) | 4 (22) | 51 (30) |
| Not examined | 6 (6) | 0 (0) | 4 (29) | 2 (11) | 12 (7) |
| Total | 109 | 29 | 14 | 17 | 170 |
| Diagnosed in non-participants | | | | | |
| Negative[b] | 44 (46) | 20 (48) | 21 (47) | 41 (34) | 126 (42) |
| Positive | 45 (47) | 15 (36) | 23 (51) | 36 (30) | 119 (39) |
| Not examined | 7 (7) | 7 (17) | 1 (2) | 43 (36) | 58 (19) |
| Total | 96 | 42 | 45 | 120 | 303 |

[a] Includes screen-detected patients who had rejected the previous screen invitation. [b] Women with DCIS included as negative.

70–74, and 15% for older women). These rates were disappointing compared with the two-county trial in Sweden, in which, among women aged 70–74 years, 72% participated after subsequent invitations (Arnesson *et al.*, 1995).

The effect of screening in the women who actually do participate may appear fairly large because the women who continue to participate have a longer life expectancy. In another study, we found a marked difference in survival of women who continued to participate at the age of 65–66 years compared with those who discontinued. The 10 year cumulative survival rates were 0.87 and 0.73 respectively (Van Dijck *et al.*, 1996). In Stockholm, similar results were reported in participants and non-participants aged 40–64 years (Lidbrink *et al.*, 1995). It is possible that participants had fewer co-existing diseases or that these were less severe. There may even be an interaction between breast cancer and certain co-existing diseases. In breast cancer patients with localised or regional disease, Satariano and Ragland (1994) found that the probability of survival decreased with an increasing number of co-existing conditions, whereas in patients with distant metastases, the 3 year survival rate did not depend on the number of other conditions. They concluded that women with severe co-existing diseases would not have a survival advantage because of early diagnosis.

One of the reasons for participation may be awareness of the presence of risk factors for breast cancer. If this is true, non-participants will be at less risk of breast cancer. The finding that the non-participant cancer rates did not increase with increasing age, in contrast to the screen-detected cancer rates and interval cancer rates, supports this hypothesis. In women over the age of 65, these non-participant cancer rates were approximately 2 per 1000 rejected invitations per annum, whereas the annual incidence of breast cancer in The Netherlands is about 3.5 per 1000 women (Netherlands Cancer Registry, 1995). In an earlier study, we also observed that the incidence of breast cancer in the non-participants was lower than would have been expected on the basis of a population without mass screening (Van Dijck *et al.*, 1996). This means that one explanation for the high incidence in elderly participants, which was 4.5 per 1000 accepted subsequent invitations per annum at ages 65+ (calculated by the summation of screen-detected cancer and interval cancer rates in Table II), may be that the women who participate at a more advanced age are at greater risk for breast cancer. However, part of the increased incidence in participants may be artificial, because some of the detected cancers may never have become clinically detectable.

As breast cancer incidence increases with increasing age, it was expected that screen-detected cancer rates and interval cancer rates would also show the same pattern. Owing to the slower growth rate (Peer *et al.*, 1993), it was expected that the ratio of screen-detected to screen-detected plus interval cancers would increase with increasing age. In the 75+ group, however, the proportion of interval cancers was relatively high. In order to find an explanation for this result, we reviewed the previous screening mammograms of 17 out of the 18 interval cancers. Two tumours (12%) had been missed at the previous screening examination; five tumours (29%) were visible in retrospect, but the signs were not specific enough for referral; and ten tumours (59%) had been radiographically occult at the previous screening. These findings are in agreement with the results of our study in 1993 and do not provide an explanation for the high interval cancer rate (Van Dijck *et al.*, 1993).

Two indicators of stage, i.e. tumour size and lymph node status, were studied. In all age groups, screen-detected tumours were the smallest. Tumours detected at repeat screening had a median size of roughly 15 mm, whereas in non-participants the median size was 25–30 mm. In all age groups there was a similar increase in the proportion of patients with negative axillary nodes due to detection at repeat screening *vs* clinical detection in non-participants. Thus, it may be concluded that, through periodic screening with mammography in women over the age of 65, breast cancer can be detected at a similar early stage as in those aged 50–64 years.

In summary, our data show that, in women aged 65 years and older, breast cancer can be diagnosed at an earlier stage by mammographic screening. This does not imply that the life expectancy of all screen-detected patients will be longer. First, a larger proportion of the screen-detected cancers may have remained undiagnosed without screening because of the slow growth rate (Peer *et al.*, 1993). Second, women of 75 have a life expectancy of 11 years and those of 85 of 6 years (Wegman, 1993). The duration of the detectable preclinical phase in women aged 70 years and older has been estimated at 4.5 years (Peer *et al.*, 1996). It is thus unlikely that many breast cancer deaths can be prevented in patients screened at age 75 years and older, but the quality of life may be increased if screening can prevent them from having to live for years with metastases.

We conclude that there is reason to continue mammographic screening until at least the age of 75 years. The beneficial effects of mammographic screening on breast cancer mortality and the quality of life may outweigh the negative side-effects until the age when life expectancy is shorter than the detectable preclinical phase of the disease.

**Acknowledgements**
We are grateful to Mr JDM Otten and Mr EP Brummelkamp for gathering and processing the data, Dr LVAM Beex for his help in ascertaining the cause of death, Ms ME Eijgenberger for constructing the figures, Mrs J Abma-Hill for her help with the English and the local authorities and general practitioners for their cooperation in the follow-up of the population. This study was supported financially by the Netherlands Health Insurance Council 'Ziekenfondsraad'.

### References

ARNESSON L-G, VITAK B, MÅNSON J-C, FAGERBERG G AND SMEDS S. (1995). Diagnostic outcome of repeated mammography screening. *World J. Surg.*, **19**, 372–378.

CHAMBERLAIN J, MOSS SM, KIRKPATRICK AE, MICHELL M AND JONES L. (1993). National Health Service breast screening programme results for 1991–2. *Br. Med. J.*, **307**, 353–356.

CHEN H-H, TABÁR L, FAGERBERG G AND DUFFY SW. (1995). Effect of breast cancer screening after age 65. *J. Med. Screening*, **2**, 10–14.

DE KONING HJ, BOER R, WARMERDAM PG, BEEMSTERBOER PMM AND VAN DER MAAS PJ. (1995*a*). Quantitative interpretation of age-specific mortality reduction from the Swedish breast cancer-screening trials. *J. Natl. Cancer Inst.*, **87**, 1217–1223.

DE KONING HJ, FRACHEBOUD J, BOER R, VERBEEK ALM, COLLETTE HJA, HENDRIKS JHCL, VAN INEVELD BM, DE BRUYN AE AND VAN DER MAAS PJ. (1995*b*). Nation-wide breast cancer screening in the Netherlands: support for breast-cancer mortality reduction. *Int. J. Cancer*, **60**, 777–780.

FLETCHER SW, BLACK W, HARRIS R, RIMER BK AND SHAPIRO S. (1993). Report of the International Workshop on screening for breast cancer. *J. Natl. Cancer Inst.*, **85**, 1644–1656.

LIDBRINK E, FRISELL J, BRANDBERG Y, ROSENDAHL I AND RUTQVIST L-E. (1995). Nonattendance in the Stockholm mammography screening trial: relative mortality and reasons for nonattendance. *Breast Cancer Res. Treat.*, **35**, 267–275.

NETHERLANDS CANCER REGISTRY. (1995). *Incidence of Cancer in The Netherlands, 1992*. Visser O, Coebergh JWW and Schouten LJ (eds). Utrecht, The Netherlands.

NYSTRÖM L, RUTQVIST LE, WALL S, LINDGREN A, LINDQVIST M, RYDÉN S, ANDERSSON I, BJURSTAM N, FAGERBERG G, FRISELL J, TABÁR L AND LARSSON L-G. (1993). Breast cancer screening with mammography: overview of Swedish randomised trials [published erratum appears in *Lancet*, **342**, 1372, 1993]. *Lancet*, **341**, 973–978.

OTTEN JDM, VAN DIJCK JAAM, PEER PGM, STRAATMAN H, VERBEEK ALM, MRAVUNAC M, HENDRICKS JHCL AND HOLLAND R. (1996). Long-term breast cancer screening in Nijmegen: the 9 rounds from 1975–1992. *J. Epidemiol. Commun. Health*, **50**, 353 – 358.

PEER PGM, VAN DIJCK JAAM, HENDRIKS JHCL, HOLLAND R AND VERBEEK ALM. (1993). Age-dependent growth rate of primary breast cancer. *Cancer*, **71**, 3547–3551.

PEER PGM, HOLLAND R, HENDRIKS JHCL, MRAVUNAC M AND VERBEEK ALM. (1994). Age-specific effectiveness of the Nijmegen population-based breast cancer-screening programme: assessment of early indicators of screening effectiveness. *J. Natl. Cancer Inst.*, **86**, 436–441.

PEER PGM, VERBEEK ALM, STRAATMAN H, HENDRIKS JHCL AND HOLLAND R. (1996). Age-specific sensitivities of mammographic screening for breast cancer. *Breast Cancer Res. Treat.*, **38**, 153–160.

SATARIANO WA AND RAGLAND DR. (1994). The effect of comorbidity on 3-year survival of women with primary breast cancer. *Ann. Intern. Med.*, **120**, 104–110.

TABÁR L, DUFFY SW AND BURHENNE LW. (1993). New Swedish breast cancer detection results for women aged 40–49. *Cancer*, **72**, 1437–1448.

VAN DIJCK JAAM, VERBEEK ALM, HENDRIKS JHCL AND HOLLAND R. (1993). The current detectability of breast cancer in a mammographic screening program: review of the previous mammograms of interval and screen-detected cancers. *Cancer*, **72**, 1933–1938.

VAN DIJCK JAAM, HOLLAND R, VERBEEK ALM, HENDRIKS JHCL AND MRAVUNAC M. (1994). Efficacy of mammographic screening of the elderly: a case referent study in the Nijmegen programme in the Netherlands. *J. Natl Cancer Inst.*, **86**, 934–938.

VAN DIJCK JAAM, VERBEEK ALM, BEEX LVAM, HENDRIKS JHCL, HOLLAND R, MRAVUNAC M, STRAATMAN H AND WERRE JM. (1996). Mammographic screening after the age of 65 years: evidence for a reduction in breast cancer mortality. *Int. J. Cancer*, **66**, 727–731.

WEGMAN ME. (1993). Annual summary of vital statistics–1993. *Pediatrics*, **92**, 743–754.

YANICK R, RIES LG AND YATES JW. (1989). Breast cancer in aging women: a population-based study of contrasts in stage, surgery, and survival. *Cancer*, **63**, 976–981.